# Proceedings of the Working Group Session on Fertility Preservation for Individuals with Gender and Sex Diversity

**DOI:** 10.1089/trgh.2016.0008

**Published:** 2016-06-01

**Authors:** Courtney Finlayson, Emilie K. Johnson, Diane Chen, Elizabeth Dabrowski, Yasmin Gosiengfiao, Lisa Campo-Engelstein, Ilina Rosoklija, Jill Jacobson, Margarett Shnorhavorian, Mary Ellen Pavone, Molly B. Moravek, Herbert J. Bonifacio, Lisa Simons, Janella Hudson, Patricia Y. Fechner, Veronica Gomez-Lobo, Rachel Kadakia, Angela Shurba, Erin Rowell, Teresa K. Woodruff

**Affiliations:** ^1^ Division of Endocrinology, Ann & Robert H. Lurie Children's Hospital of Chicago, Chicago, Illinois.; ^2^ Department of Pediatrics, Northwestern University Feinberg School of Medicine, Chicago, Illinois.; ^3^ Division of Urology, Ann & Robert H. Lurie Children's Hospital of Chicago, Chicago, Illinois.; ^4^ Department of Urology, Center for Healthcare Studies, Northwestern University Feinberg School of Medicine, Chicago, Illinois.; ^5^ Division of Adolescent Medicine, Department of Child and Adolescent Psychiatry, Ann & Robert H. Lurie Children's Hospital of Chicago, Chicago, Illinois.; ^6^ Department of Psychiatry & Behavioral Sciences, Northwestern University Feinberg School of Medicine Chicago, Chicago, Illinois.; ^7^ Division of Hematology/Oncology, Ann & Robert H. Lurie Children's Hospital of Chicago, Chicago, Illinois.; ^8^ Department of Obstetrics and Gynecology, Alden March Bioethics Institute, Albany Medical College, Albany, New York.; ^9^ Division of Endocrinology, Department of Pediatrics, Children's Mercy Hospital, University of Missouri-Kansas City School of Medicine, Kansas City, Missouri.; ^10^ Division of Urology, Seattle Children's Hospital, Department of Urology, University of Washington Seattle, Seattle, Washington.; ^11^ Department of Obstetrics and Gynecology, Northwestern University Feinberg School of Medicine, Chicago, Illinois.; ^12^ Department of Obstetrics and Gynecology, University of Michigan Medical School, Ann Arbor, Michigan.; ^13^ Division of Adolescent Medicine, Department of Pediatrics, The Hospital for Sick Children, University of Toronto, Toronto, Canada.; ^14^ Division of Adolescent Medicine, Ann & Robert H. Lurie Children's Hospital of Chicago, Chicago, Illinois.; ^15^ Department of Health Outcomes and Behavior, H. Lee Moffitt Cancer Center, Tampa, Florida.; ^16^ Division of Endocrinology, Department of Pediatrics, Seattle Children's Hospital, University of Washington Seattle, Seattle, Washington.; ^17^ Division of Pediatric and Adolescent Gynecology, Department of Obstetrics and Gynecology, MedStar Washington Hospital Center/Children's National Health System, Washington, District of Columbia.; ^18^ Division of Pediatric Surgery, Department of Surgery, Ann & Robert H. Lurie Children's Hospital of Chicago, Northwestern Feinberg School of Medicine, Chicago, Illinois.

**Keywords:** disorders of sex development, fertility, gender dysphoria, intersex, transgender

## Abstract

Children and adolescents with gender and sex diversity include (1) gender-nonconforming and transgender individuals for whom gender identity or expression are incongruent with birth-assigned sex (heretofore, transgender) and (2) individuals who have differences in sex development (DSD). Although these are largely disparate groups, there is overlap in the medical expertise necessary to care for individuals with both gender and sex diversity. In addition, both groups face potential infertility or sterility as a result of desired medical and surgical therapies. The Ann & Robert H. Lurie Children's Hospital of Chicago (Lurie Children's) gender and sex development program (GSDP) provides specialized multidisciplinary care for both transgender and DSD patients. In response to patient concerns that recommended medical treatments have the potential to affect fertility, the Lurie Children's GSDP team partnered with experts from the Oncofertility Consortium at Northwestern University to expand fertility preservation options to gender and sex diverse youth. This article summarizes the results of a meeting of experts across this field at the annual Oncofertility Consortium conference with thoughts on next steps toward a unified protocol for this patient group.

## Introduction

Oncofertility emerged over the last decade as a field that aims to preserve fertility for cancer patients undergoing treatments, including chemotherapy and radiation, which can lead to infertility.^1,2^ To expand fertility preservation to gender and sex diverse individuals, the Lurie Children's gender and sex development program (GSDP) team took the following initial steps: (1) discussed formative experiences with the leaders in the Oncofertility Consortium, (2) reviewed the limited research available on fertility in transgender and DSD patient populations, and (3) mapped the issues faced in expanding fertility preservation to individuals with gender and sex diversity. Specifically, the Lurie Children's GSDP team documented risks of infertility, the effects of medical and surgical intervention, and psychosocial concerns and ethical considerations in each population.

Although members of both the transgender and DSD communities face infertility, the reasons for fertility challenges largely differ between the two groups. Transgender youth possess inherently normal reproductive capability, but face potential infertility as a result of medical treatments intended to facilitate phenotypic transition to an affirmed gender. Pubertal suppression treatment, prescribed to youth with gender dysphoria as early as Tanner stage 2 of puberty, pauses the development of an undesired puberty, including some irreversible secondary sexual characteristics, but also prevents maturation of primary oocytes and spermatogonia to mature oocytes and sperm. Gender-affirming hormone treatment with exogenous estrogen and testosterone in adolescence may affect fertility, but the threshold at which sex steroid treatment impairs fertility is unknown.^3^

In contrast, DSD conditions are often associated with abnormal gonadal development, progressive gonadal failure over the first two decades of life, and/or abnormal hormone production, all of which can cause infertility.^3^ Furthermore, DSD conditions may be associated with elevated risk for germ cell cancer^4^; thus, traditionally, gonadectomy was recommended for some DSD conditions, in which infertility was presumed and risk of germ cell cancer was high.

**FIG. 1.**
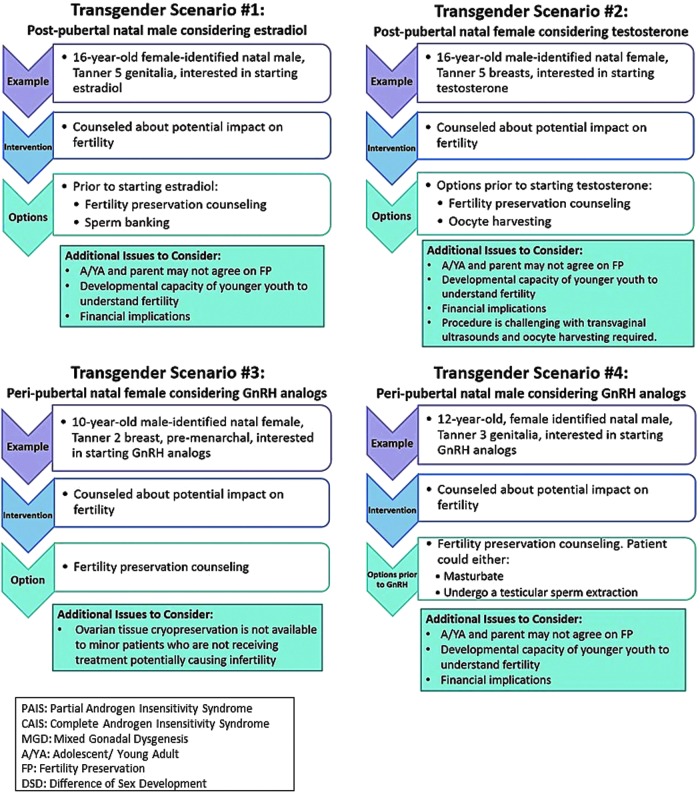
Transgender fertility preservation scenarios.

**FIG. 2.**
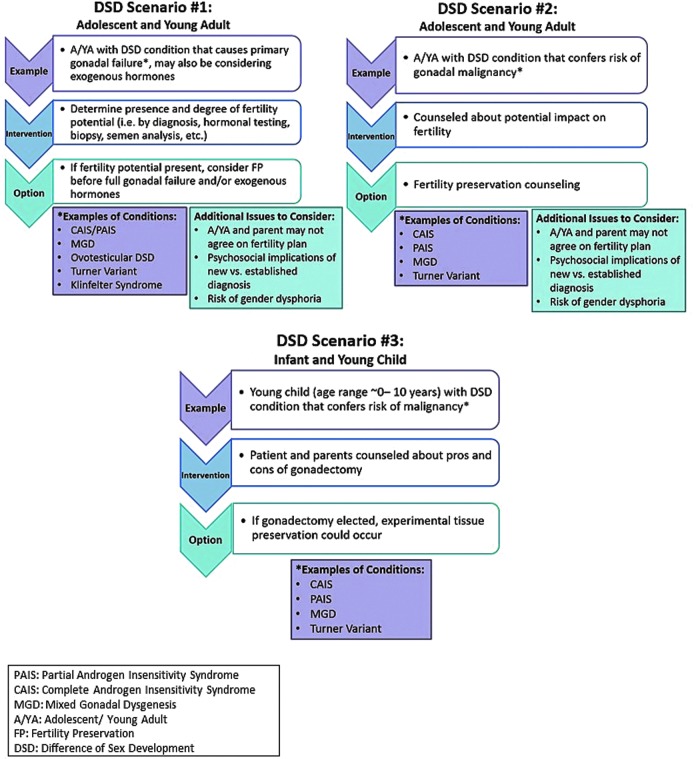
DSD fertility preservation scenarios. DSD, differences in sex development.

From these early efforts, the Lurie Children's GSDP team synthesized six representative clinical scenarios (Figs. 1 and 2) to promote discussion. Appreciating the need to gather further expertise, a working group session about fertility preservation for individuals with gender and sex diversity was convened at the November 2015 Oncofertility Consortium Meeting in Chicago, Illinois. Attendees included those with expertise in fertility preservation and/or clinical treatment of transgender and DSD patients, with foundational training in the following disciplines: bioethics, reproductive endocrinology, pediatric psychology, pediatric urology, pediatric and adolescent gynecology, pediatric endocrinology, and adolescent medicine. Each scenario in Figures 1and 2 was discussed with particular emphasis on technical needs, necessary team members, psychosocial concerns, ethical concerns, and barriers to care, including the cost of procedures. Proceedings of the session are summarized.

## Fertility Preservation Options for Postpubertal Youth

Individuals who have completed pubertal development are reproductively mature. For those with ovarian tissue, options for fertility preservation include the following: (1) embryo banking, which requires hormonal stimulation for retrieval and a suitable sperm donor, (2) oocyte banking, also requiring hormonal stimulation for retrieval, and (3) ovarian tissue cryopreservation, which requires retransplantation in the future, or *in vitro* maturation techniques, which are currently experimental.^5^ While still experimental, there have been at least 60 live births following tissue transplant and either *in vitro* fertilization with induction of oocyte maturation or natural pregnancies.^6^ For those with testicular tissue, sperm is obtained for cryopreservation through masturbation or testicular sperm extraction. Testicular tissue cryopreservation is also possible, but as with ovarian tissue cryopreservation, remains investigational.^7^

### Special considerations for transgender youth

Attendees discussed scenarios 1a–b noting that postpubertal transgender youths face specific challenges surrounding fertility preservation. First, given that the threshold at which gender-affirming hormones affect fertility is unknown, optimal timing for fertility preservation is likely before initiation of hormone treatment and, therefore, pursuing preservation procedures may delay hormone treatment. It can take several weeks from the time a patient expresses interest in fertility preservation to actually moving forward with scheduled procedures. In addition, an oocyte harvesting cycle, specifically, requires on average an additional 2 weeks and hormonal stimulation of the ovaries. Transgender individuals often experience long-standing gender dysphoria and may find delaying gender-affirming hormone treatment, even by a few weeks, distressing. Furthermore, among transmasculine individuals, hormonal stimulation of the ovaries results in increased estrogen, which patients may find unacceptable.

Second, the techniques required for preservation are invasive and may exacerbate body dysphoria, which is frequently present among transgender individuals. Oocyte harvesting requires vaginal penetration for ultrasound monitoring and for oocyte retrieval. The simplest method of sperm retrieval is by masturbation. Such penetration or stimulation of anatomy incongruent with gender identity may be particularly distressing. Third, the harvested gametes will not match gender identity. To our knowledge, no qualitative studies exist that examine whether the type of gametes to be harvested would impact an individual's choice to pursue fertility preservation. There have been documented cases of biological parenthood in transgender individuals,^8^ suggesting that congruence between gamete type and gender identity is not necessary for individuals who choose to pursue genetic parenthood.

### Special considerations for youth with differences in sex development

Review of scenarios 2 a and b elicited issues specific to postpubertal youth with DSD. As noted above, reduced inherent fertility potential is a strong possibility in some DSD conditions, thus there is no guarantee of viable oocytes or sperm. Premature gonadal failure is common; however, due to variation in timing, the window of opportunity for preservation is unpredictable. Moreover, youth and parents may be heavily burdened as they consider the complicated topic of fertility preservation while weighing the risks and benefits of potential gonadectomy to prevent germ cell cancer. Also specific to this population, many DSD conditions are known to be caused by a genetic mutation, with the possibility of transmission to offspring. As such, it is important to discuss risk for intergenerational transmission of DSD and availability of preimplantation genetic screening. As with the transgender population, gametes may not match gender identity. Anecdotally, DSD clinical care providers have expressed concern that patients and families may struggle to conceptualize the use of gametes incongruent with gender identity, but studies have not evaluated this question, thus the implications on an individual's decision to pursue fertility preservation are unknown. Finally, timing of DSD diagnosis may influence an individual's willingness to carefully evaluate options for fertility preservation. Many of these conditions are detected during adolescence, a developmental period that is critical for identity formation^9^ and during which sensitivity to privacy and emotional and cognitive functioning is particularly essential.^10^ Adolescence also marks a developmental period during which sexuality and sexual identity are explored.^11^ As such, a DSD diagnosis, particularly one in which an individual's gonads are incongruent with gender identity and assigned sex at birth (e.g., Complete Androgen Insensitivity Syndrome), may devastate an adolescent's sense of personal identity and self-esteem^10^ precluding careful consideration of options for fertility preservation.

## Fertility Preservation Options for Pre- and Peripubertal Youth

**FIG. 3.**
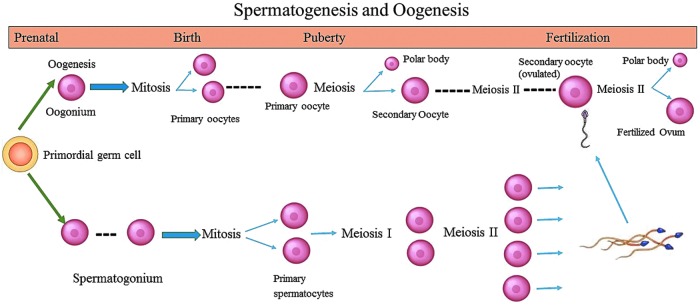
Spermatogenesis and oogenesis.

Maturation of gametes occurs during puberty, as shown in Figure 3. For individuals who have reached Tanner Stage 3–4, mature oocytes or sperm may be present, allowing preservation options similar to postpubertal youth. Individuals who have not yet reached sexual maturity, however, do not possess mature oocytes or sperm and are, therefore, limited in options for fertility preservation (see scenarios 1c,d, and 2c). Ovarian and testicular tissue may be harvested through biopsy or gonadectomy and cryopreserved. The first live birth following ovarian tissue autotransplantation from a pubertal, but premenarchal girl, was recently reported; thus, this may increasingly become an option.^12^ *In vitro* follicle growth of ovarian tissue or maturation of sperm, however, remains investigational. As such, attendees unanimously agreed that such procedures must be performed under a protocol approved by an institutional review board (IRB) at an institution with expertise in these techniques. Protocols should be specific to the population, either gender or sex diverse youth. Inclusive language, such as gonad, rather than ovary or testicle, allows for preservation of either type of tissue, regardless of gender identity and is inclusive of individuals with ovotesticular DSD, who possess both ovarian and testicular tissue.

### Special considerations for transgender youth

Considerations specific to pre- and peripubertal transgender youth with gender dysphoria include decisions about whether and when to pursue medical treatment to facilitate physical transition. Current guidelines for medical treatment of transgender youth with gender dysphoria recommend gonadotropin releasing hormone agonists (GnRHa) to pause pubertal development among early pubertal youth as early as Tanner Stages 2–3.^13^ Pubertal suppression may alleviate psychological distress related to ongoing or anticipated pubertal changes, prevent progression of secondary sexual characteristics incongruent with affirmed gender identity, allow time for further exploration of gender identity, and provide families more time for discussion and decision-making.^14^ Adolescents with persistent gender dysphoria may go on to initiate later in adolescence. An additional effect of GnRHa, however, is preventing maturation of germ cells, which could be used for biological fertility potential. Preservation of ovarian or testicular tissue using IRB-approved protocol could be pursued any time before the use of gender-affirming hormones. Alternatively, the adolescent could allow some pubertal development to mature gametes, before hormone treatment, but there was consensus that this option would likely be less desirable to the youth and antithetical to the use of GnRHa for prevention of secondary sexual characteristics. The group agreed that discussion of the fertility effects of GnRHa therapy should begin before starting treatment. The gender care provider most well-known to the individual and family should initiate and repeat these discussions. Ideally, these conversations would also take place with the individual's mental health provider during the recommended assessment of readiness for medical interventions as outlined by WPATH^15^ and Endocrine Society clinical practice guidelines.^13^

### Special considerations in youth with differences in sex development

As for postpubertal individuals with DSD, pre- and peripubertal youth with DSD may face uncertainty about inherent fertility potential and potential gonadal insufficiency. The group agreed that these questions complicate recommendations for the optimal timing of fertility preservation. Preliminary discussions may begin shortly after birth, in the case of an infant diagnosed with a DSD. Completing diagnostic testing and understanding the nature of the DSD condition can be a lengthy process of adjustment for parents, requiring patience and continued discussion. Conversation about fertility must continue in earnest if considering gonadectomy. Risk of progressive gonadal failure in early childhood, in conditions such as XO/XY Turner Syndrome and Klinefelter Syndrome, may advance the ideal timing of preservation to a younger age when primary oocytes are still present. Again, the group recognized the possibility of transmitting a genetic condition to offspring, highlighting the need to weigh this risk and consider options for preimplantation genetic screening.

## Team Approach

The group recommends a team approach for discussing and implementing fertility preservation procedures in gender and sex diverse individuals. Working group attendees offered experience with a range of institutional practice and team members. This led to an understanding that a proper fertility preservation team at one institution may be different than at another, based on the experience and availability of providers. For example, there can be overlap in roles of an endocrinologist and adolescent medicine specialist in the care of transgender youth or in surgeons' roles given varying experience with fertility preservation. In general, however, participants agreed that the team should include members with training in disciplines as shown in Table 1.

**Table 1.** **Team Approach to Fertility Preservation**

Discipline	Role on fertility preservation team
Psychology/social work	Facilitate discussion of desire for biological fertility. Assess individual's capacity for medical decision-making, family dynamics, and transgenerational desire for fertility. Provide support for individuals struggling to cope with potential infertility.
Endocrinology	Assess gonadal function and likelihood of biological fertility potential. Discuss fertility-related implications of medical transition treatments in transgender youth.
Adolescent medicine	Discuss fertility-related implications of medical transition treatments in transgender youth.
Urology	Counsel about sperm preservation. Perform testicular biopsy, TESE. Assess internal anatomy and constitution of gonadal tissue. Perform gonadal biopsy or gonadectomy.
Pediatric surgery	Assess internal anatomy and constitution of gonadal tissue. Perform gonadal biopsy or gonadectomy.
Obstetrics/gynecology	Assess internal anatomy and constitution of gonadal tissue. Perform gonadal biopsy or gonadectomy.
Reproductive endocrinology	Counsel about and perform ovarian stimulation and oocyte retrieval.
Ethics	Evaluate individual's ability to assent/consent. “Arbitrate” in situations, in which parents and youth may disagree on decisions for fertility preservation.
Financial counseling	Discuss financial implications of procedures and storage, often not included in insurance coverage.
Genetic counseling	Discuss risks of transmission of condition to offspring and role of preimplantation genetics.
Fundamental reproductive science	Develop new technologies for measuring fertility loss and restoring endocrine and fertility in high-risk cases.

TESE, testicular sperm extraction.

In addition, the group agreed that including other stakeholders will be essential in guiding the direction of this field. Members of this working group are part of the National physicians cooperative (NPC), a national interdisciplinary group of specialists, which provides fertility preservation clinical services to cancer patients, including basic scientists, allied health professionals, and physician groups (urology, reproductive endocrinology, general surgery, obstetrics/gynecology, and hematology/oncology). The NPC was born from needs associated with cancer patients, but now extends to incorporate other fields as fertility threats are identified. The subset of the NPC for gender and sex diverse individuals should seek input from youth affected by these conditions and their parents, as well as advocate for unique and important perspectives from those outside the medical field.

## Ethical Concerns

In discussing the options for fertility preservation in gender and sex diverse youth, the group raised many ethical concerns. First, as in many dilemmas in the pediatric population, issues arise regarding a patient's ability to participate meaningfully in medical decision-making (i.e., issues related to assent versus consent). While fertility decisions ideally are made by an individual in adulthood, postponing decision-making may result in missing an optimal window during which fertility preservation is most likely to be successful. Second, the fact that parents, as the legal guardian and proxy for the youth, make decisions for their child can raise concerns, especially if there are divergent views. For example, parents' wishes may differ from those of their child or may differ between parents sharing legal decision-making rights. Third, providers may exert pressure due to their own personal biases regarding issues such as alternative options for parenting, whether to pursue fertility preservation, and the role of preimplantation genetic screening. Fourth, there is great concern about the implications of encouraging “false hope” for fertility potential. As reviewed, fertility potential in many of these conditions is unknown and *in vitro* maturation of cryopreserved prepubertal tissue is experimental, with no guarantee for successful future use of preserved immature gametes. Thus, the group suggested using the term “cryopotential,” rather than cryopreservation, to denote storage of immature tissue, where the potential for future use is still under development. Finally, ownership of the biological material must be established and documented before preservation. Adult individuals specify the fate of the material in the event of their death: donation to research, to another individual, or that the material be destroyed. Embryos made with a partner's gametes belong to the partner. In the pediatric population, current policies specify that in the event of death, tissue is either destroyed or donated for research.

## Technical Requirements

Success in fertility preservation is a complicated process, dependent on multidisciplinary expertise and specific technical abilities. Thus, the group determined that fertility preservation for gender and sex diverse individuals should be offered at centers that can provide both clinical and technical expertise, with a designated team leader. In addition to the team specified in Table 1, pathology expertise is also important to examine and process the tissue and an assisted reproductive technology and andrology laboratory to further process the specimens. A patient navigator is extremely helpful to guide patients through the process, and research assistants are important for regulatory support in opening and maintaining protocols, as well as collecting data. One of the roles that the NPC played in the development of the oncofertility field was a series of guidelines and protocols that could be shared and adapted by practitioners even as larger specialty societies were developing guidelines. Having team members work together across disciplines catalyzes the work.

## Barriers to Care

In discussing other potential barriers to care, participants identified geographic and financial concerns. The scarcity of fertility preservation centers limits some individuals' ability to pursue fertility preservation, particularly if travel to a center is too burdensome. Furthermore, the cost of the preservation process itself may be prohibitive. Potential fees include those for harvesting of tissue or retrieval of gametes (operating room, surgery, and anesthesia fees), consultation with fertility preservation specialists, hormones to stimulate oocyte production, tissue processing fees, and shipping of gametes or tissue to a storage facility and long-term storage. Insurance coverage for such procedures is extremely limited and state specific, although the Oncofertility Consortium has worked to address this issue.^16^ Table 2 provides estimated procedure and storage costs at Lurie Children's, as well as the likelihood of insurance coverage. It is important to note that these cost data are institution specific and include some negotiated pricing. The notations about insurance coverage reflect the experience in oncofertility and may be found to be different in the gender and sex diverse population. Long-term storage remains the individual's responsibility and need-based financial assistance is offered by some storage facilities. Limited philanthropic support for storage now exists for oncology patients and is a potential avenue for defraying some costs for gender and sex diverse individuals.

**Table 2.** **Cost of FP Techniques: Experience of Lurie Children's Hospital Oncofertility Team**

Technique	Cost	Insurance coverage
Oocyte cryopreservation	$5000–$10,000	No
Ovarian tissue cryopreservation^a^ (consultation, oophorectomy, and freezing of ovary)	$9000–$20,000	Variable
Semen analysis	$375	Usually
Freezing semen	$350	No
TESE	$8000	Variable
Testicular tissue Cryopreservation^a^ (consultation, testicular biopsy, or orchiectomy)	$5500	Usually
Infectious disease testing	$240	Yes
Shipping to storage facility	$215	No
Yearly storage	$275 ($75 discount based on financial need)	No

^a^ Experimental.

## Summary

The inaugural meeting of the Gender and Sex Diversity Fertility Working Group set the stage for a new field in fertility preservation. This report synthesizes the current state of the field, gaps in knowledge, and goals to address moving forward (Table 3). Ultimately, the goal of improved care for these populations will be achieved through collaborative interdisciplinary work.

**Table 3.** **Action Items for the Gender and Sex Diverse Working Group**

Immediate action items
Initiate a formal review of the bioethical concerns in each population. Of particular importance is the need to address the ethical concerns related to the experimental nature of some techniques to preserve fertility potential and the lack of data about fertility potential in these patients.
Invite patient advocates from each community to join the working group and provide their crucial perspective.
Build transgender and DSD fertility information into the current Oncofertility website.
Long-term research needed to advance the field
Multicenter extension of ongoing Lurie study to determine the presence and quality of germ cells in gonads of patients with disorders in sex development.
Study gonads in adult transgender patients undergoing sex reassignment surgery to evaluate the effect of hormone treatment on gonadal function and fertility.
Qualitative study to determine attitudes toward fertility in patients with complete androgen insensitivity syndrome, with a focus on discordance between gonadal type and gender identity.
Multicenter trials to preserve gonadal tissue and potential fertility in prepubertal gender and sex diverse individuals.

DSD, differences in sex development.

## Abbreviations Used

DSD: differences in sex development
GnRHa: gonadotropin releasing hormone agonists
GSDP: gender and sex development program
IRB: institutional review board
NPC: National physicians cooperative
TESE: testicular sperm extraction
